# A Note on Scalar-Gradient Sharpening in the Stable Atmospheric Boundary Layer

**DOI:** 10.1007/s10546-020-00516-x

**Published:** 2020-04-10

**Authors:** J. Antoon van Hooft

**Affiliations:** Department of Geoscience and Remote Sensing, Delft University of Technology, Delft, The Netherlands

**Keywords:** Dipolar vortex, Direct numerical simulation, Scalar fronts, Stable boundary layer, Temperature ramps

## Abstract

The scalar front generated by the horizontal self advection of a dipolar vortex through a modest scalar gradient is investigated. This physical scenario is an idealization of the emergence of strong temperature ramps in the stable atmospheric boundary layer. The proposed mechanism is discussed and a two-dimensional analogy is studied in depth using direct numerical simulation. More specifically, the scalar-gradient sharpening is investigated as a function of the Reynolds number. It appears that the process of gradient sharpening at large-eddy scales may be challenging for turbulence-resolving methods applied to the stable-boundary-layer regime.

**Electronic supplementary material:**

The online version of this article (10.1007/s10546-020-00516-x) contains supplementary material, which is available to authorized users.

## Introduction

**Fig. 1:**
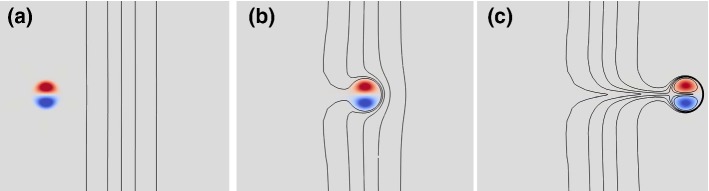
The process of scalar-front generation by dipolar self advection at Re = 800 (see Sect. 2). **a** A Lamb–Chaplygin dipolar vortex (vorticity field in colour, with red, positive values and blue, negative) is initialized together with a passive tracer field (black isolines). **b** The vortex structure moves to the right through the original scalar front, which is modest in magnitude. **c** A much sharper scalar front emerges at the vortex edge. The five isolines indicate 10%, 30%, 50%, 70%, and 90% of the total horizontal scalar field inversion. See the supplementary material for a video depicting the evolution of these snapshots (Electronic Supplementary Material 1 ‘Re800-overview.mp4’)

Coherent scalar-gradient sharping is typically not associated with turbulent flows. As such, the emergence of sharp temperature fronts forms an intriguing aspect of the stable atmospheric boundary layer (SBL). This note concerns the generation of such fronts by dipolar-vortex structures as is illustrated in Fig. 1, with a visualization of a two-dimensional (2D) direct numerical simulation (see Sect. 2 for details). A dipolar vortex is initialized and advects itself towards a passive scalar front with a moderate spatial gradient (black isolines). The flow structure propagates due to the entrainment of the vortices by each other. As the system evolves, the isolines of the scalar tracer field converge near the edge of the vorticity patches (see also, Eames and Flór 1998). Hereby the local gradients in the scalar field sharply increase and the dissipation of the scalar-field variance is enhanced.

Scalar fronts in SBL flows have both been observed and modelled. A conditionally-sampled analysis of large-eddy-simulation results by Sullivan et al. (2016) indicates that sharp temperature fronts in the SBL coincide with the dynamics of coherent vortex structures. A horizontal cross-section of such structures revealed a dipolar structure, where two oppositely-signed patches of vorticity are located in each other’s vicinity. In their study, such vortex columns are identified as the legs of hairpin vortices commonly found in three-dimensional (3D) wall-bounded turbulent flows (Hommema and Adrian 2003; Adrian 2007). Furthermore it is well known that dipolar vortex structures can easily emerge in stratified flows, as demonstrated by the laboratory experiments of van Heijst and Flór (1989), Voropayev et al. (1991), and Flór and Van Heijst (1994). Here, vertical motions are limited due to the stratification that may suppress isotropic flow at large scales, which, for sufficiently strong stratification, gives rise to quasi-2D flow. While the identification of coherent vortex structures in the atmosphere from field observations is challenging, and typically relies on indirect footprints of such structures (Cuxart et al. 2002; Barthlott et al. 2007), indications of so-called ‘pancake vortex structures’ have been reported. Here, the characteristic motions of the air have a much larger horizontal extent compared with the vertical flow scales (Galperin et al. 2007; Mahrt 2009; Sun et al. 2015), hinting at vertically-restricted dynamics.

Based on laboratory data, numerical results, and field observations, this study concerns the aforementioned, heavily idealized scenario to describe the generation of large-scale temperature fronts in SBL flows. The self-advective property of dipolar-vortex structures makes them a prime suspect for the generation of scalar fronts. In order to study this process in more depth, the problem is isolated from its meteorological context. It turns out that the scalar-field-gradient amplification in the present surrogate scenario is only limited by the diffusivity of momentum and the scalar quantity. A useful integral measure for the strength of a scalar front is the integral of the squared absolute gradient, which is directly related to the dissipation rate of the scalar-field variance. As such, we concern ourselves here with the amplification of the scalar dissipation by dipolar self-advection as a function of the medium’s diffusivity.

Computer simulations need to idealize (or parametrize) the complexity of the atmosphere’s dynamics in order to reduce a physical problem to a numerical one. The advantage is that this facilitates controlled studies on the interactions between the relevant processes. Even though the present set-up focuses on a candidate building block of the SBL structure, and has little direct resemblance to the full flow reality, the author believes that the detailed analysis yields interesting results for the atmospheric-boundary-layer community. I stress the need for future field observations of turbulence and temperature structures. With promising advancements in observational technologies, such as distributed temperature sensing (Thomas et al. 2012; Lapo et al. 2019) and thermal imaging (Grudzielanek and Cermak 2015, 2018), there is hope for revelations on this topic.

## Numerical Set-Up

**Fig. 2:**
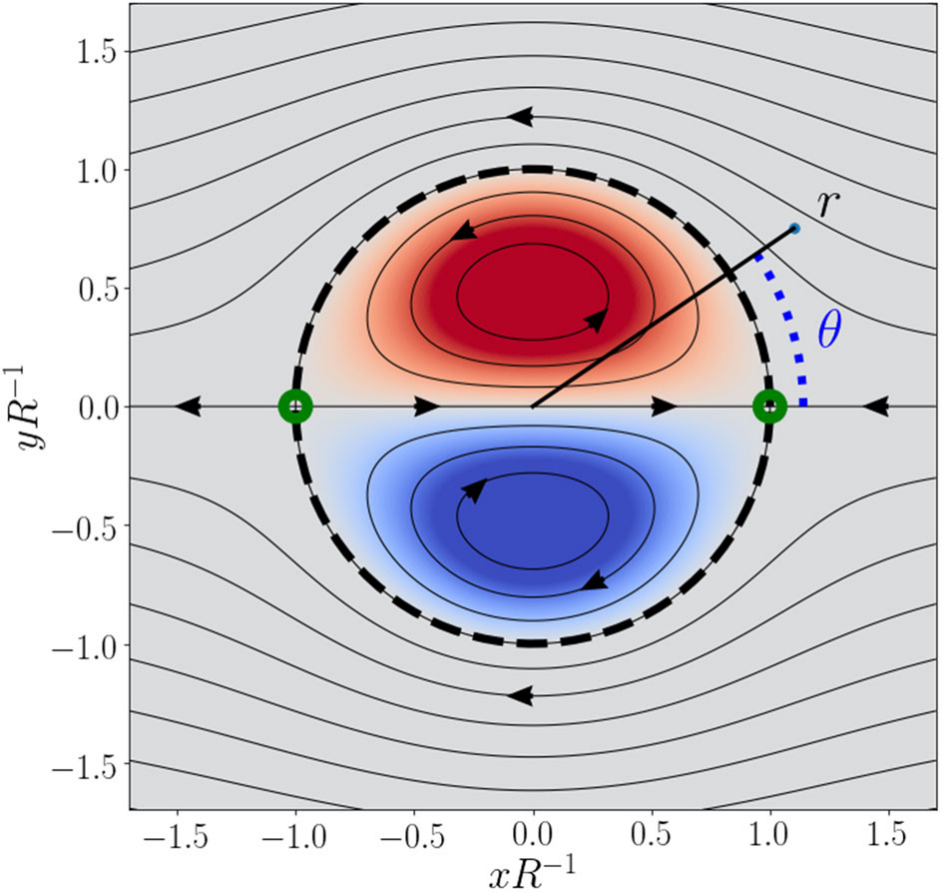
The flow structure of the Lamb–Chaplygin dipolar vortex, showing the streamlines ($\psi = \mathrm {constant}$) evaluated in the co-moving frame of reference, with the colours indicating the vorticity field ($\omega = \nabla ^2 \psi$). The dashed line marks the separatrix, a streamline that encloses the vortex structure. Arrow heads are drawn to indicate the local flow direction and green circles annotate the locations of the stagnation points (see text)

In order to model the self advection of a 2D vortex structure, the steady dipolar-vortex model for inviscid flow proposed by H Lamb and SA Chaplygin is employed (Meleshko and Van Heijst 1994), which describes a vortex structure with a circular radius of size *R* that propagates without deformation through an otherwise irrotational fluid at a flow speed *U*. The streamfunction $\psi$ of the so-called Lamb–Chaplygin dipole, moving in the positive *x* direction, in the co-moving frame of reference is given in cylindrical coordinates ($r, \theta$, see Fig. 2) by

1 $$\begin{aligned} \psi = {\left\{ \begin{array}{ll} \frac{-2 U J_{1}(kr)}{kJ_{0}(kR)}\mathrm {sin}(\theta ) , &{} \text {for } r < R, \\ U\left( \frac{R^2}{r}-r\right) \mathrm {sin}(\theta ), &{} \text {for } r \ge R, \end{array}\right. } \end{aligned}$$

where $J_0(x)$ and $J_1(x)$ are the zeroth and first Bessel function of the first kind for a dummy variable *x*, respectively, and *k* is an inverse length scale with a value such that $kR = 3.83...$, the first non-trivial zero of $J_1(x)$ (i.e. $J_1(3.83...) = 0$).

As a model for a temperature field with an initially moderate horizontal front, a passive tracer field *s* is used, which gradually changes from low to high values for increasing *x*, according to

2 $$\begin{aligned} s = A\ \mathrm {tanh}\left( \frac{x}{\sigma } \right) , \end{aligned}$$

with *A* an amplitude-of-*s* scale, ‘$\mathrm {tanh}$’ the hyperbolic tangent function, and $\sigma$ is a length scale of the initial scalar transition. Here, $\sigma = 2R$ is chosen and the vortex structure is initially placed at $x = -5R$, where $s \approx -A$, such that it advects towards the right-hand side (r.h.s.) of the initial scalar transition, where $s \approx A$.

A generic feature of dipolar vortices is the so-called separatrix: a streamline in the co-moving frame that encloses the vortex structure and, thereby, the dipole entrains the fluid inside this region. Figure 2 depicts the circular separatrix (of radius *R*) of the Lamb–Chaplygin dipole model and reveals the presence of two stagnation points; two streamlines at $\psi = 0$ coincide. At these stagnation points, the scalar gradient would increase to a singularity if it were not affected by the medium’s diffusivity. As such, the flow is considered to be affected by the fluid’s viscosity $\nu$, and the evolution of the tracer field *s* is subject to the diffusivity $\kappa$. The following dimensionless parameters can be constructed from the parameters $R, U, \nu$ and $\kappa$,

3 $$\begin{aligned} \hbox {Re} = \frac{UR}{\nu }, \end{aligned}$$

known as the Reynolds number and,

4 $$\begin{aligned} \hbox {Pr} = \frac{\kappa }{\nu }, \end{aligned}$$

known as the Prandtl number. The latter is set to unity (Pr = 1) and the evolution of tracer-field properties is studied as a function of the Reynolds number Re. The range of Re values is restricted to $50 \le \hbox {Re} \le 6400$, which covers the limits from minimal gradient sharpening to the apparent asymptotic scaling for large Re values (see Sect. 3).

The set of equations for fluid motion (the Navier–Stokes equations), together with the advection–diffusion equation for the tracer field *s*, are solved in the frame of reference that is co-moving with the initialized dipolar vortex ($u_{\mathrm {trans}} = U$). The evolution of the system is solved until $t_{\mathrm {end}} = 15RU^{-1}$ in a large square domain ${\mathcal {D}}$ with size $L_0^2 = 25R\times 25R$. The vortex structure is initialized in the domain centre so that the exact location of the domain’s boundaries has little influence on the results presented herein. The bulk flow (i.e. $u_{\mathrm {trans}}$) is directed to the left direction inside a free-slip channel with a no-flux condition for the field *s* at the walls. The left-hand-side (l.h.s.) boundary facilitates outflow conditions by setting the normal derivative to zero for the velocity components ($u_x, u_y$) and the tracer field *s*. Inflow occurs at the r.h.s. boundary ($x = x_r$) and applies Dirichlet conditions to $u_x(x_r) = -u_{\mathrm {trans}}$ and $s(x_r) = A\ \mathrm {tanh}(\frac{x_r+tu_{\mathrm {trans}}}{\sigma })$.

Numerically, the adaptive quadtree-grid solver for the Navier–Stokes equations within the freely-available Basilisk code is employed (see www.basilisk.fr and Popinet 2015). The adaptive-grid approach is attractive as it consistently chooses a grid resolution based on the fidelity of the discrete representation of the relevant fields, lifting this non-trivial burden from the model user. Further, it focuses the computational resources towards the regions in space and time where they are most required. The numerical schemes are identical to those used in earlier work on adaptive turbulence-resolving simulations (van Hooft et al. 2018a). The grid-element sizes are adaptively refined and coarsened based on the representation of the second-order polynomial content in the scalar field. Refinement-criterion values for the scalar field ($\zeta _s = 0.02A$) and for the velocity-component fields ($\zeta _{u_i} = 0.02U$) resulted from a convergence study, and aim to balance the accuracy of the results versus the required computational effort (see van Hooft et al. 2018b for a detailed example). The maximum resolution is not explicitly limited; the algorithm increased it to $R\varDelta _{\mathrm {min}}^{-1} = 655.36$ for the run where Re = 6400. This mesh-element size corresponds to that of a $16384 \times 16384$-cell equidistant grid.

The computer code is available at: www.basilisk.fr/sandbox/Antoonvh/gradient-sharpening.c.

## Results and Implications

Figure 1 depicts the global evolution of the flow for Re = 800. As the vortex structure moves to the right, the isolines converge at the dipole’s separatrix where a large absolute gradient in the tracer field is generated, and defined by

5 $$\begin{aligned} \Vert \varvec{\nabla } s\Vert = \sqrt{\left( \frac{\partial s}{\partial x}\right) ^2 + \left( \frac{\partial s}{\partial y}\right) ^2}. \end{aligned}$$

A global measure for the strength of the scalar front is the domain ${\mathcal {D}}$ integral of $\Vert \varvec{\nabla } s\Vert ^2$

6 $$\begin{aligned} \epsilon = \iint \limits _{{\mathcal {D}}} \Vert \varvec{\nabla } s\Vert ^2 \mathrm {d}O. \end{aligned}$$

**Fig. 3:**
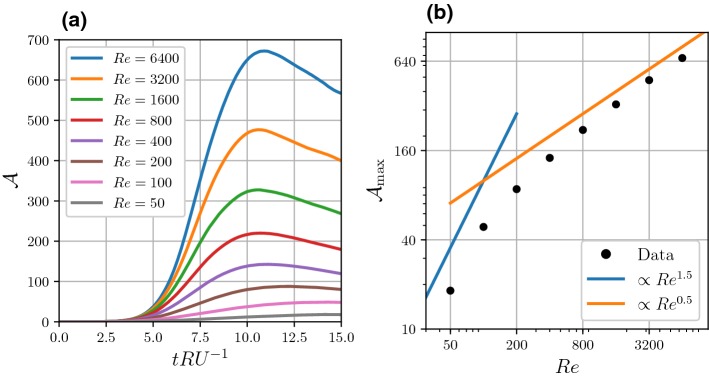
**a** The evolution of the scalar-gradient-amplification measure ${\mathcal {A}}$ (see Eq. 8), and **b** the maximum value of ${\mathcal {A}}$ plotted against the Reynolds number Re on logarithmic scales

In order to quantify the gradient amplification due to the dipole self-advection with a measure that is insensitive to the exact domain size, we take two steps to modify $\epsilon$:

1. $\epsilon$ is ‘corrected’ with its initial value,
  7 $$\begin{aligned} \epsilon _{\mathrm {DP}} = \epsilon - \epsilon _{t = 0}. \end{aligned}$$
2. The amplification ${\mathcal {A}}$ is defined by scaling $\epsilon _{DP}$ with the initial $\epsilon$ value per unit length *R* in the *y* direction, giving
  8 $$\begin{aligned} {\mathcal {A}} = \frac{\epsilon _{DP}}{RA\int \nolimits _{\mathrm {left}}^{\mathrm {right}} \left( \frac{\mathrm {d}}{\mathrm {d}x}\mathrm {tanh}\left( \frac{x}{\sigma } \right) \right) ^2 \mathrm {d}x}\equiv \epsilon _{DP}\frac{25}{\epsilon _{t=0}}, \end{aligned}$$

where the factor 25 is specific for the chosen length of the domain in the *y* direction ($L_0R^{-1} = 25$). The time evolution of the amplification ${\mathcal {A}}(t)$ is plotted for various Reynolds numbers in Fig. 3a. For each of the studied Reynolds numbers, there exists a temporal maximum corresponding to the dipolar vortex being on the r.h.s. of the transition at a time where the diffusivity of the scalar *s* has not yet started to smooth the scalar gradient. As such, Fig. 3b plots the maximum value ${\mathcal {A}}_{\mathrm {max}}$ against Re, illustrating that for high Re values (say, $\hbox {Re} > 800$), ${\mathcal {A}}_{\mathrm {max}}$ scales with Re according to

9 $$\begin{aligned} {\mathcal {A}}_{\mathrm {max}} \propto \hbox {Re}^{0.5}, \end{aligned}$$

which follows the 2D Prandtl boundary-layer theory (Kundu et al. 2015, chap. 10) and is a consequence of the inviscid scaling of dissipation (Tennekes et al. 1972). For the high Reynolds numbers, the flow field is not significantly affected by the viscosity, as the Lamb–Chaplygin model is a steady solution in the limit of inviscid flow. For the lower Reynolds numbers (i.e. $\hbox {Re} < 800$), both the momentum and scalar fields are affected by their respective diffusivities ($\nu$ and $\kappa$). Apparently, this results in a stronger sensitivity to the value of Re compared with the high-Reynolds-number regime.

We conclude from this idealized scenario that 2D dipolar-vortex columns form an effective mechanism for the generation of scalar fronts.

Since temperature fronts are a relevant large-scale feature of the SBL, and indeed, both observations (e.g., Balsley et al. 2003) and numerical simulations show that coherent vortex structures such as hairpins (Adrian 2007) are the rule rather than the exception in the SBL, the corresponding dynamics should be captured properly by numerical models for the SBL. The results indicate that the severity of the temperature front is sensitive to the value of the Reynolds number or the effective Reynolds number equivalent. For large-eddy simulation, a diffusive subgrid-scale closure is typically invoked to model the flow at the unresolved scales, of which there are a large variety of formulations, and as such, the modelling of flow problems as discussed herein is likely to be sensitive to the details of the chosen closure.

**Fig. 4:**
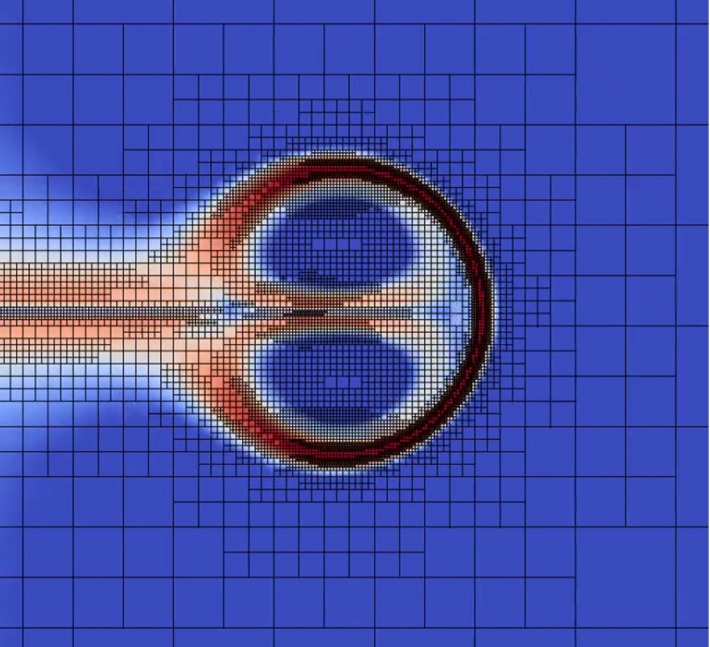
A snapshot of the adaptive quadtree-grid structure and the $\Vert \nabla s\Vert$ field at $t = 10RU^{-1}$ for the simulation with Re = 800. Blue and red colours indicate regions with low and high values for the scalar gradient ($\Vert \nabla s\Vert$), respectively. See the supplementary material for a movie depicting the evolution of this snapshot (Electronic Supplementary Material 2 ‘Re800-grid.mp4’)

The present example of the 2D surrogate system clearly shows that scalar fronts (e.g., of temperature) may emerge that are much smaller in width than the eddy size itself, and so this challenge was approached by studying a reduced-dimensional problem with respect to the realistic SBL, and an adaptive grid was employed to focus the computer resources. Figure 4 depicts the $\Vert \nabla s\Vert$ field and the corresponding adaptive-grid structure used for the computations at $t = 10 RU^{-1}$ for Re = 800. For this dynamical system, a high resolution is required to capture the thin scalar boundary layer at the vortex-structure edge.

## Electronic supplementary material

Below is the link to the electronic supplementary material.

Supplementary material 1 (mp4 743 KB)

Supplementary material 2 (mp4 127 KB)
